# Plants accumulate abscisic acid after *Ralstonia solanacearum* infection for enhanced dehydration tolerance and plant resistance

**DOI:** 10.3389/fpls.2025.1566215

**Published:** 2025-06-05

**Authors:** Yao Wang, Min Yan, Anbin Wang, Xingjun Ma, Weiqiang Tian, Ying Liu, Liquan Zhu, Wei Ding, Shili Li

**Affiliations:** ^1^ Laboratory of Natural Products Pesticides, College of Plant Protection, Southwest University, Chongqing, China; ^2^ Yibin Tobacco Company of Sichuan Province, Yibin, China; ^3^ College of Agronomy and Biotechnology, Southwest University, Chongqing, China; ^4^ Zunyi Branch Company of Guizhou Tobacco Company, Zunyi, China

**Keywords:** abscisic acid, plant water content, induced resistance, tobacco bacterial wilt, *R. solanacearum*

## Abstract

Plants will display typical dehydration and wilting symptoms after *Ralstonia solanacearum* infection. Many studies have shown that abscisic acid (ABA) has been implicated in wilting, but the role of ABA after *R. solanacearum* infection remains largely unexplored. The plant water potential and endogenous ABA content of tobacco plants were investigated after *R. solanacearum* infection, and we assessed the preliminary mechanisms and control effect of exogenous ABA on tobacco bacterial wilt. Here we show that *R. solanacearum* can reduce leaf water content (LWC) and leaf water potential (Ψ_leaf_) and promote the accumulation of ABA on leaves. Notably, foliar spraying 0.78 mg/L ABA could alleviate the wilting by increasing Ψ_leaf_ and decreasing the stomatal size, stomatal conductance (Gs), and transpiration rate (Tr). Furthermore, 0.78 mg/L ABA application promoted plant growth, reduced the colonization of *R. solanacearum*, increased the activities of defense enzymes, upregulated the expression of JA/ET-related and ROS-related genes, and suppressed the expression of SA-related gene. Moreover, 0.78 mg/L ABA could reduce the incidence of tobacco bacterial wilt, with the control efficiency reaching up to 54.94% at 11 dpi, significantly higher than that of benzothiazole (BTH) with 19.33%. Our findings provided a new result for exogenous ABA controlling tobacco bacterial wilt by reducing water loss and enhancing plant resistance.

## Highlights

- *R. solanacearum* promoted plant water loss.
- Plants accumulated ABA to reduce water loss caused by *R. solanacearum* infection.
- Foliar spraying 0.78 mg/L ABA promoted plant growth and enhanced the activity of defense enzymes.
- Spraying 0.78 mg/L ABA upregulated the expression of JA/ET and ROS-related genes.
- Spraying 0.78 mg/L ABA delayed the progression of tobacco bacterial wilt.

## 1 Introduction

Plant diseases are important determinants of food yield losses and quality reduction, which threaten food security and social stability (Savary et al., 2019). The second most destructive bacterial plant pathogen *R. solanacearum* is widespread in tropical, subtropical, and warm temperate areas and infects 450 plant species in over 54 families, such as tobacco, potato, pepper, tomato, and eggplant, with production losses of solanaceae crops of up to 15%–70% or even extinction in severe cases (Aslam and Mukhtar, 2023a; Genin, 2010; Mansfield et al., 2012). At present, the engineered nanoparticles (DA@CMCS-NPs), crop rotation (maize or lily rotation), biological control agents (*Trichoderma harzianum*), and plant-derived natural products (Hydroxycoumarins) have been studied, but these technologies have been less widely applied on the control of the disease (Niu et al., 2016; Wang et al., 2023; Wu et al., 2017; Yang et al., 2016; Yaseen et al., 2025). It is well known that *R. solanacearum*, as a species complex, has high variability and strong adaptability, owing to differences in soil types, temperature, moisture, and other edaphic factors of various areas (Shahbaz et al., 2015; Aslam and Mukhtar, 2023b). *R. solanacearum* invaded plant roots from wounds (or root tips) to rapidly multiply in the xylem (Milling et al., 2011). Subsequently, producing exopolysaccharide (EPS) promoted the colonization of *R. solanacearum* but also hindered water transport in plants, which ruptured the canal due to excessive hydrostatic pressure, causing wilting and plant death (Aslam and Mukhtar, 2024). Interestingly, we found that the early wilting caused by *R. solanacearum* infection could be naturally restored under sufficient plant water. Therefore, plant water regulation is particularly important. It is well known that the plant water potential is a physiological indicator of water regulation capacity (Boanares et al., 2020; Spinelli et al., 2017). The more water at high plant water potential will be transported to water-deficient cells, and the ability to absorb water is stronger (Kannenberg et al., 2022). In addition, the higher Ψ_leaf_ promoted nutrient uptake, and the lowest Ψ_leaf_ was conducive to occurrence and spread of coffee brown spot (Vilela et al., 2022). *Pseudomonas syringae* could sense water potential (-1.6 to -2.2 MPa), and a low water potential was able to decrease the number of endophytic bacterial population (Wright and Beattie, 2004). It can be seen that water potential is a limiting factor for the growth of pathogens. Water potential can regulate the osmotic pressure of plant tissues to maintain water balance, which is closely linked to ABA.

ABA is a major actor in regulating the water shortage of plants; it also stimulates stomata closure and inhibits stomata opening, mitigating the decline in water potential and preventing plant death due to water deficiency (Mphande et al., 2021; Yari Kamrani et al., 2022). Stomatal conductance controlled by ABA on different time scales suppresses water loss (Franks et al., 2017), and the timing and rate of stomatal closure vary considerably among species before early xylem cavitation, with some species closing their pores rapidly to maintain a high water potential, while others close their pores slowly as water potential decreases (Brodribb and McAdam, 2011; Martin-StPaul et al., 2017). In terms of activating plant resistance, ABA stimulates the expression of defense-related genes and, subsequently, the biosynthesis of protective compounds, e.g., dehydrins (Schwarzenbacher et al., 2020; Sun et al., 2021; You et al., 2010). Simultaneously, in the responses to biotic stresses, ABA was in cooperation with jasmonic acid (JA) for responding to the attack of herbivorous insect. In contrast, JA interacts with ethylene upon necrotroph attack, thereby improving plant disease resistance (Adie et al., 2007; Rekhter et al., 2019). Exogenous ABA application increased the activity of tomato leaf defense enzymes (PAL, PPO, and POD) and defense genes (GLU, PR1, PPO, SOD, and POD genes) expression to resist tomato black spot disease (Song et al., 2011). Moreover, among different resistant varieties, the amount of ABA was higher in KCB-1 resistant variety than in CB-1 susceptible variety 24 h after *R. solanacearum* infection (Shi et al., 2022). On the contrary, ABA inactivated SOD and CAT activities, inhibited the accumulation of reactive oxygen species (ROS) and the JA pathway, and promoted rice black streak dwarf virus infection (Xie et al., 2018). However, there are not a lot of reports about the changes of ABA and water potential on the development of tobacco bacterial disease and the mechanism of exogenous ABA-induced disease resistance in tobacco after infection.

Therefore, the purposes of this study were to clarify the effect of ABA-mediated Ψ_leaf_ and plant immunity against *R. solanacearum*. Thus, we investigated the variation of plant water potential (Ψ_root_, Ψ_stem_, and Ψ_leaf_) in tobacco plant after *R. solanacearum* infection and measured the ABA content and Ψ_leaf_ of resistant–susceptible tobacco cultivar. Meanwhile, we analyzed the effect of ABA content in plant on the disease by foliar spraying Na_2_WO_4_, investigated the optimal concentration and preliminary mechanism of ABA inducing plant resistance to the disease, and studied the effect of ABA on plant growth, defense enzyme activities, and defense-associated genes. This study will further promote the development and application of ABA and will also provide a new idea for the management of tobacco bacterial wilt.

## 2 Materials and methods

### 2.1 Materials

*R-solanacearum* CQPS-1, the plant pathogen, was stored by Natural Products Pesticide Laboratory (Chongqing, China), and it was grown in B media (nutrient-rich media) at 30°C for 12 h. Abscisic acid (ABA, 99%) was purchased from Shanghai Yuanye Biotechnology Company. Seeds of K326 and Yunyan 87 were provided by Yuxi Zhong Yan Tobacco Seed Co., Ltd.

### 2.2 Plant cultivation and inducting method

The selected seeds were dipped in 75% ethanol for 30 s and then 30% H_2_O_2_ for 10 min. Then, the seeds were placed on a substrate of Danish Pinchotop charcoal soil, in which the culture condition was a relative humidity of 60%, a day/night temperature of 28°C/20°C, and 14/10 h light/dark cycle. The seed cultures of Yunyan 87 and K326 were the same, which could be used after growing into three-leafed tobacco seedlings.

The foliar spraying method was used to induce tobacco resistance. Briefly, the prepared solutions were sprayed evenly on the leaf surface for a total of two times. Afterward, the 6-week-old unwounded tobacco plants were inoculated with 10 mL of fresh *R. solanacearum* suspension (OD_600_ = 0.1, ≈10^8^ CFU/mL).

### 2.3 Water content of different plant parts

To investigate the plant water content with different disease grades, we measured the root, stem, and leaf water content (RWC, SWC, and LWC) of Yunyan 87. Firstly, the method of inoculation with *R. solanacearum* was referred to in Section 2.2. After 3 days, collected tobacco seedlings with different disease grades (grades 0, 1, 2, 3, and 4, respectively) were weighed to measure the fresh weight. Each disease grade was 10 tobacco seedlings (the stem and root length were approximately 4.4–4.5 and 8.9–9.1 cm, and the number of leaves was three maximum leaves. Each treatment had three biological replicates, each comprising 10 plants. Next, all of the fresh samples were transferred to an oven at 105°C for 30 min and then were placed in 80°C for 48 h, and the dry weight was measured (Zhou et al., 2021). The plant water content was calculated by the following Equation 1:


(1) WC(%)=100×(FW−DW)/FW

where FW is fresh weight and DW is dry weight. The different disease grades were defined as follows (Han et al., 2021): 0, no wilt symptom; 1, 1%–25% of leaves wilted; 2, 26%–50% of leaves wilted; 3, 51%–75% of leaves wilted; 4, 76%–100% of leaves wilted.

### 2.4 Determination of plant water potential

To clarify the relationship between the different disease levels and plant water potential (Ψ_leaf_, Ψ_stem_, and Ψ_root)_, a PSYpro instrument, dew point hydrodynamics, was used to measure the plant water potential (Shelden et al., 2017), with minor modifications. Briefly, after the occurrence of the disease, leaf, stem, and root of tobacco seedlings were harvested. The plant with different disease levels (grades 0, 1, 2, 3, and 4, respectively) had the same size of round leaves (6 mm in diameter) taken with a hole punch. In addition, stem segment with thickness of 0.05 mm and root with 1 cm were cut off separately from tobacco stem and root and then quickly put into the sample chamber to investigate Ψ_leaf_, Ψ_stem_, and Ψ_root_. Each treatment consisted of three biological replicates, each comprising three plants. The Ψ_leaf_ of susceptible tobacco cultivar (Yunyan 87) and resistant tobacco cultivar (K326), respectively, was determined as detailed above.

### 2.5 Observation of leaf stomata after *R. solanacearum* infection

The observation of leaf stomata referred to a method reported in previous studies (Wang et al., 2023). Briefly, leaf samples (0.6 cm × 0.6 cm) of different disease grades (grades 0, 1, 2, 3, and 4) were immediately transferred onto a 1.5-mL EP tube with 1.2 mL 2.5% glutaraldehyde solution at 4°C for 24 h. Each treatment consisted of three biological replicates, each comprising three plants. Next, all samples were rinsed with 0.1 M PBS buffer (pH 6.8) four times (10 min each time) and dehydrated in 2 mL ethanol solution with mass concentrations of 10% to 100%. Finally, the leaf samples were freeze-dried, tiled in conductive adhesive, and observed by SEM (ApreoC, ThermoFisher).

### 2.6 ABA content measurement

To identify the changes of ABA content in plant leaves in different disease levels, firstly, the method of inoculation with *R. solanacearum* was referred to in Section 2.2. Meanwhile, leaf samples of different disease levels were taken. The ABA content was extracted and determined using the Shanghai KAIB ABA biological kit, which was the method of enzyme-linked immunosorbent Assay (ELISA) (Yang et al., 2001), with minor modifications. Briefly, leaf tissues weighing 0.1 g were transferred to liquid nitrogen for complete grinding. Then, 0.9 mL of PBS buffer (0.01 M, pH = 7.4) was added, followed by homogenization and centrifugation at 5,000 r for 10 min at 4°C. After centrifugation, the supernatant was removed and stored at -20°C. Each treatment consisted of three biological replicates, each comprising five plants. The OD_450_ was measured by 1500 model full-wavelength enzyme labeling instrument (Thermo, USA), and the regression curve was drafted using the standard concentration and the corresponding OD_450_. Subsequently, each sample concentration was determined by the curve.

### 2.7 Assessment of ABA on tobacco bacterial wilt

To obtain the best concentration of foliar spraying ABA application, we used a pot experiment to assess the controlling effect on tobacco bacterial wilt (Wang et al., 2023). Briefly, ABA dissolved in DMSO was diluted with DI water to mass concentrations of 0.38, 0.78, 1.56, 3.13, and 6.26 mg/L. Next, 0.1% DMSO was used for negative control (CK) and BTH for positive control. Each treatment consisted of biological replicates, each comprising 10 plants. Finally, disease index (DI) was scored 3 days after inoculation, and the mean of the DI was calculated relative to defense efficiency with the following Equation 2:


(2) Control efficiency (%)=[(CK−H)/CK]×100

where H represents the disease index for treatment and CK represents the same for the control group.

### 2.8 Effect of Na_2_WO_4_ on tobacco bacterial wilt

To further identify whether ABA content in plants affects disease occurrence, it was assessed by spraying ABA synthesis inhibitor (Na_2_WO_4_). Briefly, 0.5 g/L Na_2_WO_4_ was uniformly sprayed on the tobacco leaves. Next, 0.78 mg/L ABA and DI water (CK) were used as the control groups. After the treatment was done twice, *R. solanacearum* suspension (OD_600_ = 0.1) was inoculated by root irrigation at 10 mL per plant. Each treatment consisted of three biological replicates, each comprising 10 plants. DI water was used for the control group.

### 2.9 Transpirational pull and photosynthesis measurement

To further clarify the impact of ABA on transpirational pull and photosynthesis after inoculation with *R. solanacearum*, we investigated the stomatal conductance (Gs), transpiration rate (Tr), net photosynthetic rate (Pn), and intracellular CO_2_ concentration (Ci), with minor modifications. Briefly, ABA spray application and inoculation with *R. solanacearum* were referred to in Section 2.2. Leaf samples were sampled at 1, 3, and 5 days after inoculation with *R. solanacearum*. Next, Gs, Tr, Pn, and Ci were tested, respectively, by a portable photosynthesizer (USA, LI-6200) from 7:00 to 9:00 p.m. in a greenhouse under LED light conditions. The measurements were repeated three times.

### 2.10 Effect of ABA application on *R. solanacearum* infection

To assess whether ABA affects the movement of *R. solanacearum*, we measured the reproduction of *R. solanacearum* in tobacco after ABA and Na_2_WO_4_ treatments. DI water was used for the control group (CK). Briefly, the method of spraying ABA and inoculation with *R. solanacearum* were referred to in Section 2.2 and Section 2.7. At 3 days after inoculation, the tobacco stem base and roots were collected. Next, the samples were sterilized with 75% ethanol for 1 min, followed by two washes with sterile water. Afterward, the samples were sufficiently ground with sterile water and collected by centrifugation at 8,000 rpm for 3 min. Then, the obtained bacterial suspensions were gradient-diluted 10^1^, 10^2^, 10^3^, 10^4^, and 10^5^ times, respectively, and the number of *R. solanacearum* colonized at the stem base and roots, respectively, was determined by plate counting method (Yang et al., 2024). Each concentration was six biological replications, each comprising three plants. Finally, the plate was placed in the incubator at 30°C for 48 h, and the number of single colonies was recorded and calculated.

### 2.11 Effect of ABA on tobacco growth

ABA is a recognized plant hormone that affects plant growth. To define the effect of different concentrations ABA on tobacco growth, ABA concentrations of 0.38, 0.78, 1.56, 3.13, and 6.26 mg/L were configured with sterile water, respectively, and the method of foliar spraying ABA was performed according to Section 2.2 under non-inoculation. Each concentration has three biological replications, each comprising 10 plants. Then, the agronomic traits of tobacco (dry weight, fresh weight, plant height, and leaf area) were measured on the 10th day after treatment, and leaf area was calculated by the following Equation 3:


(3) $$\text{Leaf area }\left({\text{cm}}^{2}\right)=0.6345\times [L_{1}\left(\text{cm}\right)\times L_{2}\left(\text{cm}\right)]$$

where *L*_1_ is the maximum leaf length, and *L*_2_ is the maximum leaf width. The value 0.6345 is a parameter determined (Xiao et al., 2024).

### 2.12 Measurement of defense enzyme activities

To clarify the effect of exogenous spraying ABA on defense enzyme activities in tobacco. We measured the enzyme activities as described in the past report (Wang et al., 2024). Firstly, 0.1 g of fresh leaves after foliar spraying 0.78 mg/L ABA was taken at 10 min, 1 h, 6 h, 12 h, and 24 h, respectively, and then rapidly stored in liquid nitrogen for measurement. Furthermore, 0.1% DMSO served as the control group. Each treatment had biological replications, each comprising three plants. The PAL, SOD, POD, and PPO enzyme activities were extracted using a kit (Suzhou Grise Biotechnology Co., Ltd.) and measured by using UV1000 spectrophotometer (Shanghai Tianmei Scientific Instrument Co., Ltd.) at 290, 450, 470, and 420 nm, respectively.

### 2.13 RT-PCR analysis

The methods of ABA foliar application and in obtaining samples (Yunyan 87) were the same as in Section 2.6. Total RNA in leaves was extracted using an RNA kit (Solarbio, Shanghai), and cDNA was synthesized by a cDNA synthesis kit (Evo M-MLV, Solarbio). The cDNA obtained was amplified with the q-PCR kit, and the primers are shown in **Supplementary Table S1**. The reaction conditions were as follows: 20 μL reaction system, pre-denatured at 95°C for 30 s, then denatured at 95°C for 5 s, and annealed at 60°C for 30 s, for a total of 40 circulation. The relative gene expression was calculated by using the 2^-ΔΔct^ method (Li et al., 2021; Wang et al., 2019; Xie et al., 2018). Each treatment consisted of three biological replications, each comprising three plants.

### 2.14 Data analysis

Excel 2016 was used for data organization, GraphPad Prism 8.0.2 for graphing, and SPSS 16.0 for analysis by Tukey’s test or *t*-tests (*p < 0.05, **p < 0.01, ***p < 0.001, ****p < 0.0001; ns, not significant). All experiments were repeated three times, and representative pictures were selected for display.

## 3 Results

### 3.1 Plant water content will decrease after *R. solanacearum* inoculation in plants

**Figure 1:**
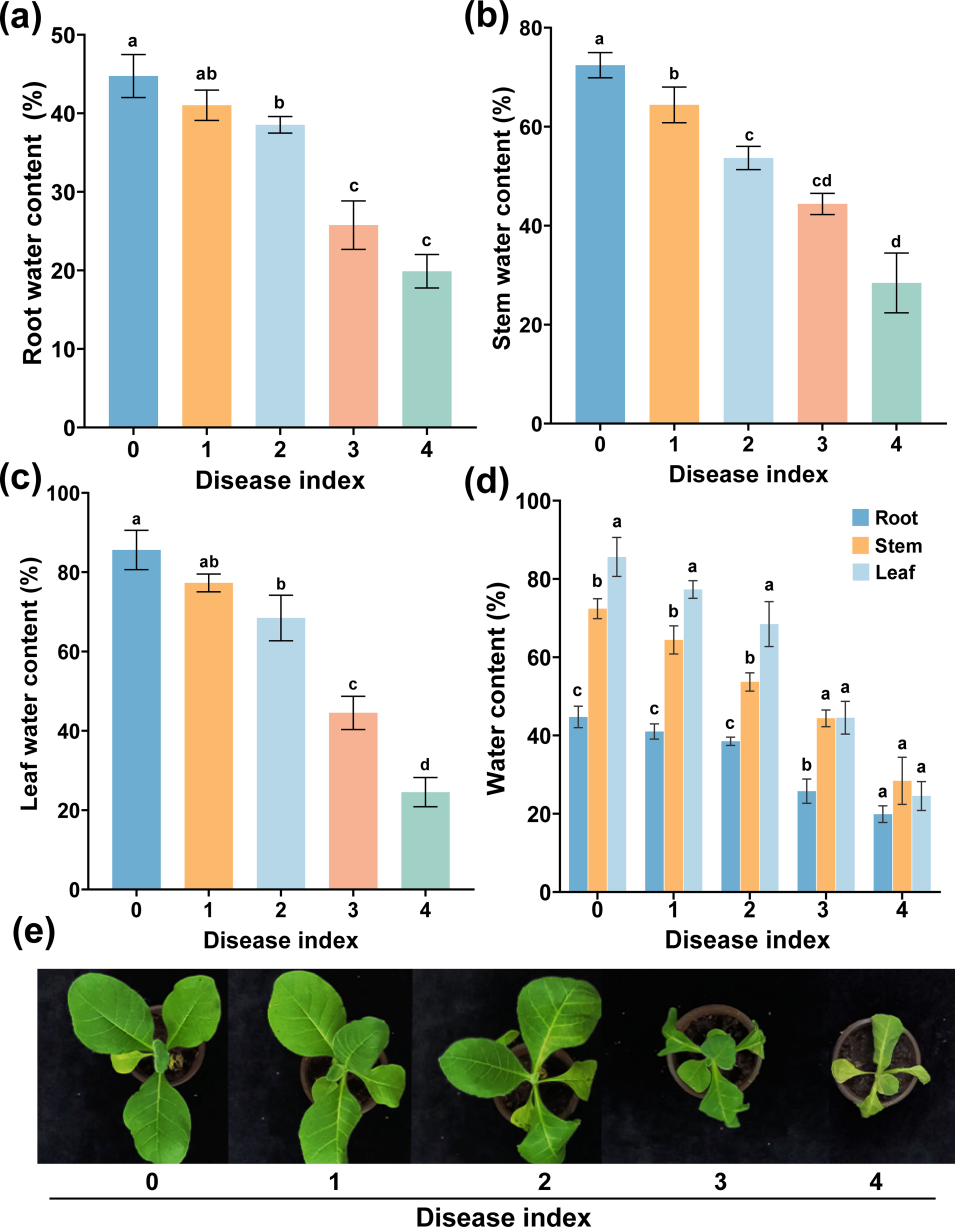
Water content of different plant parts at different disease indexes. Water content of root **(a)**, stem **(b)**, and leaf **(c)** from grades 0 to 4 and **(d)** water content of different tissues. **(e)** The symptoms of different disease index. The data are shown as mean ± standard deviation (SD). The bars with lowercase letters indicate statistically significant differences by Tukey’s test (*p* < 0.05).

Water is essential for maintaining the health and growth of plants. Water in plants could affect the infection of plant-pathogenic bacteria to the host plant (Wang et al., 2015). As shown in **Figures 1a–c**, the plant water content (PWC) of the grades 0 to 4 decreased dramatically. The PWC of grade 0 was significantly higher than that of other disease grades, and the PWC of grade 4 was the lowest, and the root water content (RWC), stem water content (SWC), and leaf water content (LWC) decreased with the increase of disease index (DI). However, except for the grade 4, the size of water content was LWC > SWC > RWC (**Figure 1d**), which decreased by 61.08%, 43.98%, and 24.85%, respectively. In conclusion, PWC decreased with the increase of DI, and the LWC decreased the most.

### 3.2 Plant water potential will decrease after *R. solanacearum* inoculation in plants

**Figure 2:**
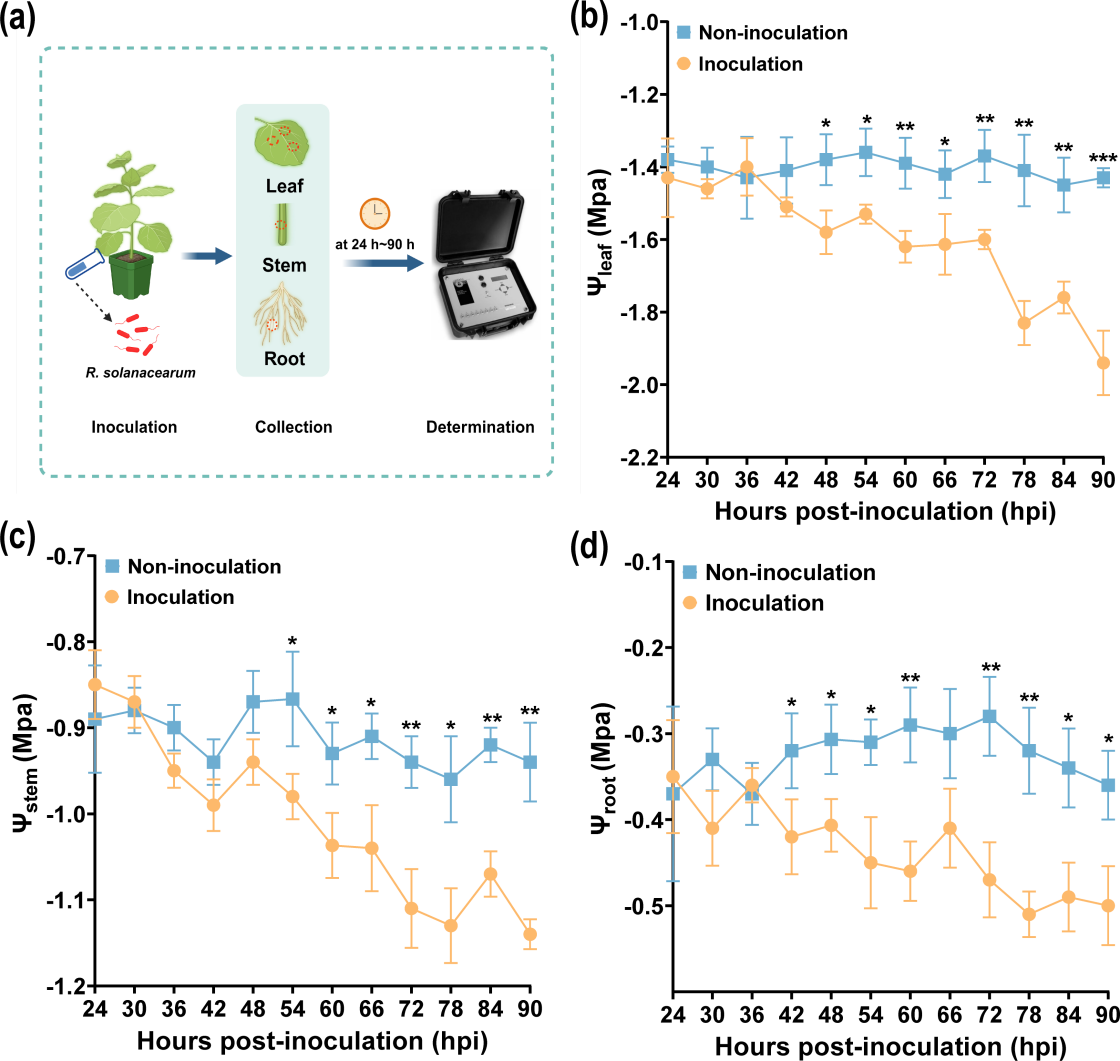
Variations of plant water potential after *R. solanacearum* inoculation. **(a)** Schematic drawing of measured Ψ_stem_, Ψ_root_, and Ψ_leaf_. **(b-d)** Variations of Ψ_leaf_, Ψ_stem_, and Ψ_root_, respectively. An asterisk (*) indicates a statistically significant difference between inoculation and non-inoculation by *t*-tests. The data are shown as mean ± SD. *p < 0.05, **p < 0.01, ***p < 0.001.

Plant water potential (PWP) is a reflection of PWC, and the low (negative) PWP can cause xylem embolism and the death of a plant (Choat et al., 2012). Based on the variation of PWC, the leaf water potential, stem water potential, and root water potential (Ψ_leaf_, Ψ_stem_, and Ψ_root_) were measured using a PSYpro dew point water potential (Wescor, USA) after *R. solanacearum* infection (10 mL/per plant, OD_600 nm_ = 0.1). As shown in **Figure 2a**, the method of measured PWC at different time points was described. The Ψ_leaf_, Ψ_stem_, and Ψ_root_ all sharply declined after inoculation from 24 to 90 h, and the Ψ_leaf_, Ψ_stem_, and Ψ_root_ of non-inoculated samples showed an upward and downward fluctuation trend (**Figures 2b–d**). In addition, the Ψ_leaf_, Ψ_stem_, and Ψ_root_ of non-inoculated samples, after 42 h, were higher than that of the inoculated ones. The Ψ_leaf_ showed significant changes in PWC after inoculation from 24 to 90 h. Interestingly, in **Figure 2b**, the Ψ_leaf_ of the non-inoculated sample was significantly higher than those inoculated at 90 h (*p* < 0.001). The results showed that PWC will decrease after *R. solanacearum* infection, and Ψ_leaf_ decreased the most, which was similar to the change of LWC.

### 3.3 The stomatal aperture of leaves decreased first and then increased in *R. solanacearum-*inoculated plants

**Figure 3:**
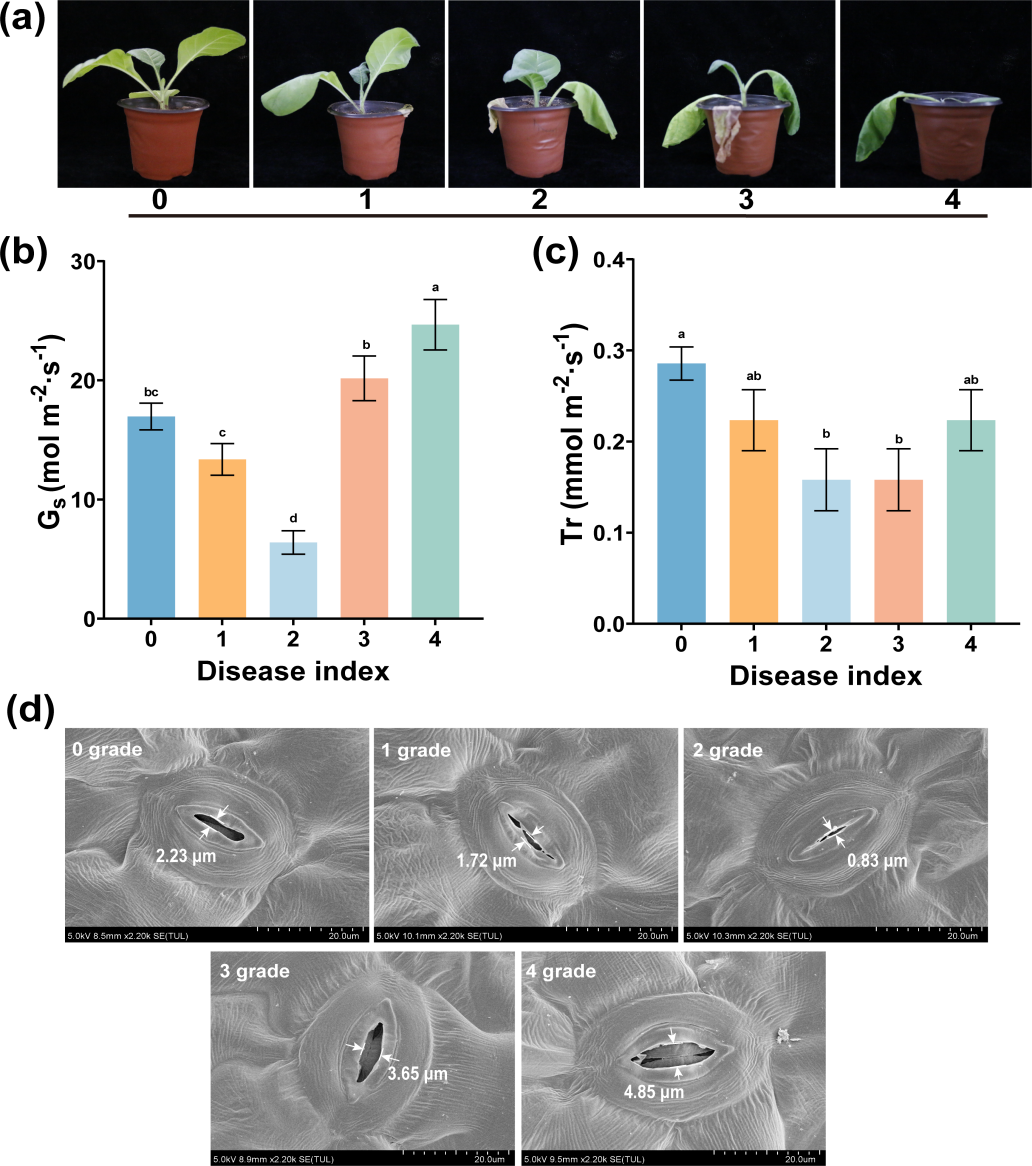
Leaf stomata in the lower epidermis of different disease levels. **(a)** Wilting symptoms of different disease levels, **(b)** stomatal conductance, Gs, and **(c)** transpiration rate, Tr. **(d)** Stomatal size for different disease levels. The data are shown as mean ± SD. The bars with lowercase letters indicate statistically significant differences by Tukey’s test (*p* < 0.05).

Shown in **Figure 3a** are plant leaves in which the wilting symptom appeared (at 3–5 dpi) after *R. solanacearum* infection. However, this early wilting has similar symptoms to that when there is natural water shortage in plants. To further study the symptom at different disease levels, we measured the *Gs*, *Tr*, and stomatal aperture after *R. solanacearum* infection. As shown in **Figures 3b, c**, from grades 0 to 4, the values of *Gs* and *Tr* showed a trend of first decreasing and then increasing, and the *Gs* and *Tr* of grade 2 were significantly lower than those of other disease grades (*p* < 0.05). Specifically, the *Gs* and *Tr* of healthy plants were 16.967 mol m^-2^ s^-1^ and 0.286 mmol m^-2^ s^-1^, respectively, which were significantly higher than the 13.367 mol m^-2^ s^-1^ and 0.223 mmol m^-2^ s^-1^ of diseased plants (grade 1). Moreover, the stomatal width across different disease levels was consistent with the trends observed in *Gs* and *Tr*, with the stomatal width in grade 2 being the minimum compared to grades 1, 3, and 4 as shown in **Figure 3d**. This shows that the Gs, Tr, and stomatal width will decrease after *R. solanacearum* infection, thereby indirectly reducing plant water loss.

### 3.4 Endogenous ABA regulates Ψ_leaf_ to counteract water loss caused by *R. solanacearum*

**Figure 4:**
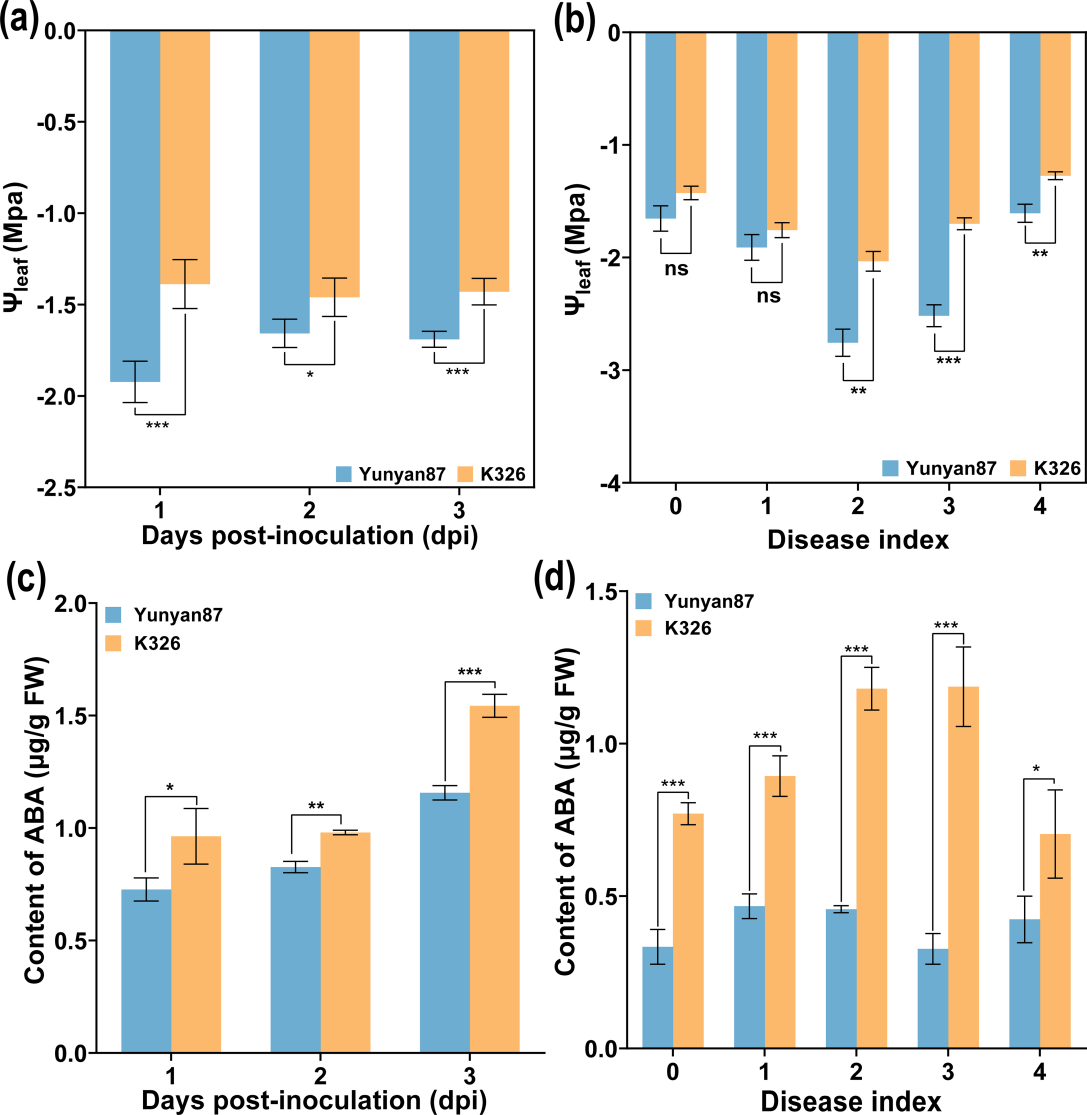
Variations of Ψ_leaf_ and ABA content of different varieties after *R. solanacearum* inoculation. **(a)** Ψ_leaf_ of different time, **(b)** Ψ_leaf_ of different disease levels, **(c)** content of ABA at different times, and **(d)** content of ABA at different disease levels. The data are shown as mean ± SD. An asterisk (*) indicates a statistically significant difference between Yunyan87 and K326 by *t*-tests. *p < 0.05, **p < 0.01, ***p < 0.001. ns, not significant.

Compared to Ψ_root_ and Ψ_stem_, the variation of Ψ_leaf_ varied greatly. To further analyze whether there were similar changes in Ψ_leaf_ among different tobacco varieties after *R. solanacearum* inoculation, the Ψ_leaf_ of susceptible tobacco cultivar (Yunyan 87) and resistant tobacco cultivar (K326), respectively, at different times and disease levels was measured. As shown in **Figure 4**, the Ψ_leaf_ of K326 was higher than that of Yunyan 87. At 1–3 days post-inoculation (dpi), the Ψ_leaf_ of K326 did not change much, while that of Yunyan 87 showed an increasing trend from 1 to 2 dpi (**Figure 4a**). At the same time, both Ψ_leaf_ of Yunyan 87 and K326 showed a downward and then upward trend during the process of disease index rising from 0 to 4, and grades 2–4 showed a significant difference (**Figure 4b**). It is known that ABA phytohormone plays a critical role in plants to respond to drought stress (Jamnická et al., 2019). Based on this, the ABA content of Yunyan 87 and K326, respectively, after *R. solanacearum* infection were investigated. As shown in **Figure 4c**, the ABA content in leaves was significantly higher in K326 compared to Yunyan 87 during 1–3 dpi, and the ABA content in the leaves of grades 0 to 4 were also significantly higher in K326 than in Yunyan 87 (**Figure 4d**). The ABA content of K326 exhibited a pattern of initially increasing and then decreasing. In addition, in Yunyan 87, the ABA content also followed a similar pattern (**Figure 4d**). The Ψ_leaf_ of tobacco plants (K326 and Yunyan 87) was regulated by endogenous ABA after *R. solanacearum* infection. It could be seen that the increase of Ψ_leaf_ and endogenous ABA may be in response to the wilting caused by *R. solanacearum*.

### 3.5 0.78 mg/L ABA was optimal concentration against tobacco bacterial wilt

**Figure 5:**
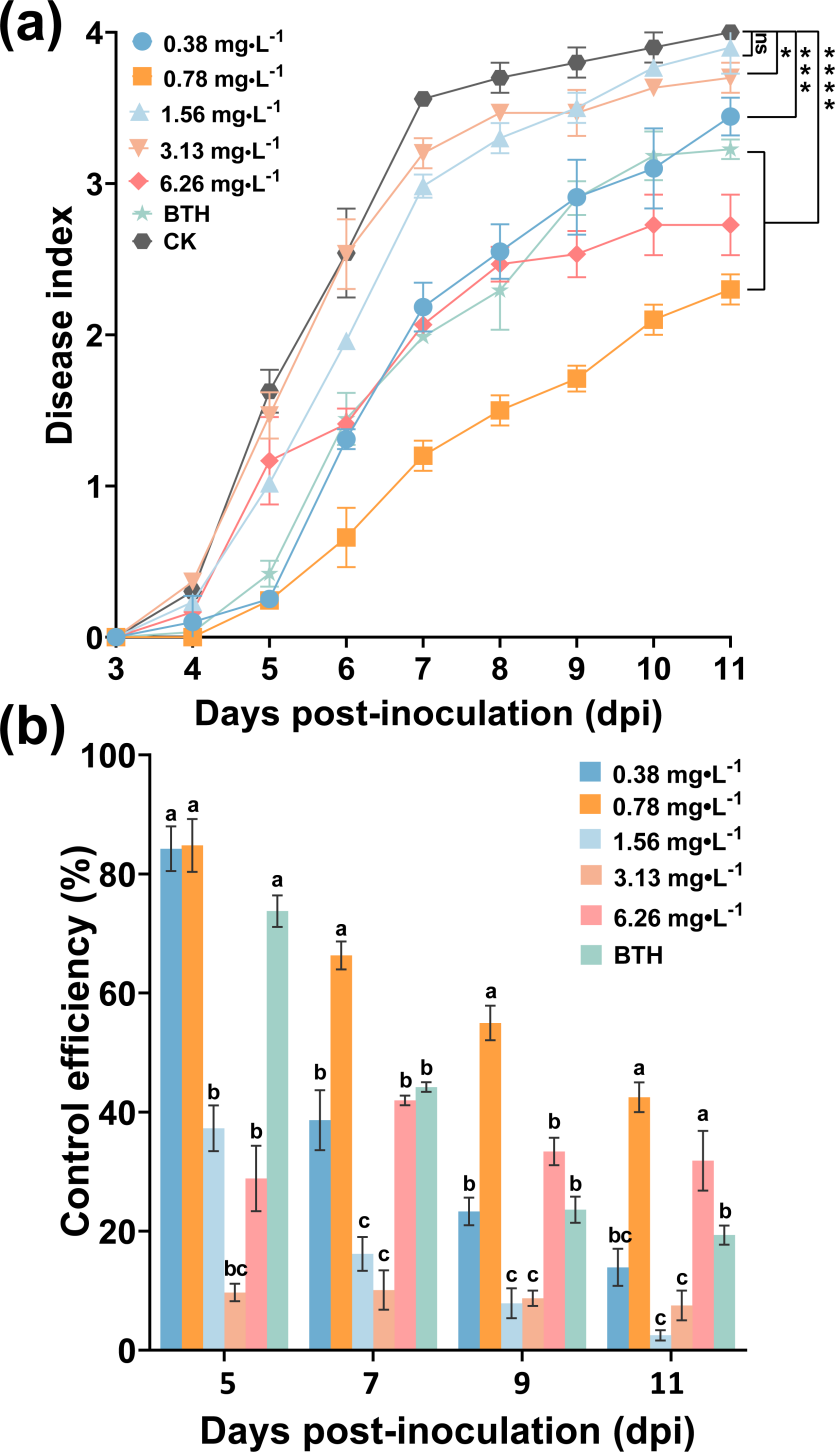
Control efficiency of foliar spraying ABA on tobacco bacterial wilt. **(a)** Disease index after treatment with ABA, BTH, and CK (DI water). **(b)** Control efficacy of ABA and BTH. The data are shown as mean ± SD. An asterisk (*) indicates a statistically significant difference between ABA and CK by *t*-tests **(a)**. *p < 0.05, **p < 0.01, ***p < 0.001, ****p < 0.0001. ns, not significant. The bars with lowercase letters indicate statistically significant differences by Tukey’s test (*p* < 0.05).

To obtain the optimal concentration of ABA that induces a tobacco plant’s resistance to *R. solanacearum*, ABA concentrations at 0.38, 0.78, 1.56, 3.13, and 6.26 mg/L were assessed. As shown in **Figure 5**, foliar spraying ABA provided a better control effect on tobacco bacterial wilt and alleviated the occurrence of the disease. ABA at concentrations from 0.38 to 6.26 mg/L had a lower disease index than CK. At 11 dpi, the disease index (DI) of ABA treatment at 0.78 and 6.26 mg/L were 2.30 and 2.73, respectively, both lower than BTH being 3.23 and markedly lower than that of CK being 4 (*p* < 0.05) (**Figure 5a**). Specifically, there were large differences between different concentrations of ABA in **Figure 5b**. In 5 days, the control efficacy of ABA (0.78 mg/L) was slightly higher than that of the BTH, the control efficacy of which was 84.80% and 73.35%, respectively. At 11 dpi, the control efficacy of foliar spraying ABA was 54.96%, which was significantly higher than BTH at 19.33%. These results indicated that ABA at 0.78 mg/L was the optimal concentration for induced tobacco resistance to *R. solanacearum*.

### 3.6 Foliar spraying Na_2_WO_4_ promoted the occurrence of tobacco bacterial wilt

**Figure 6:**
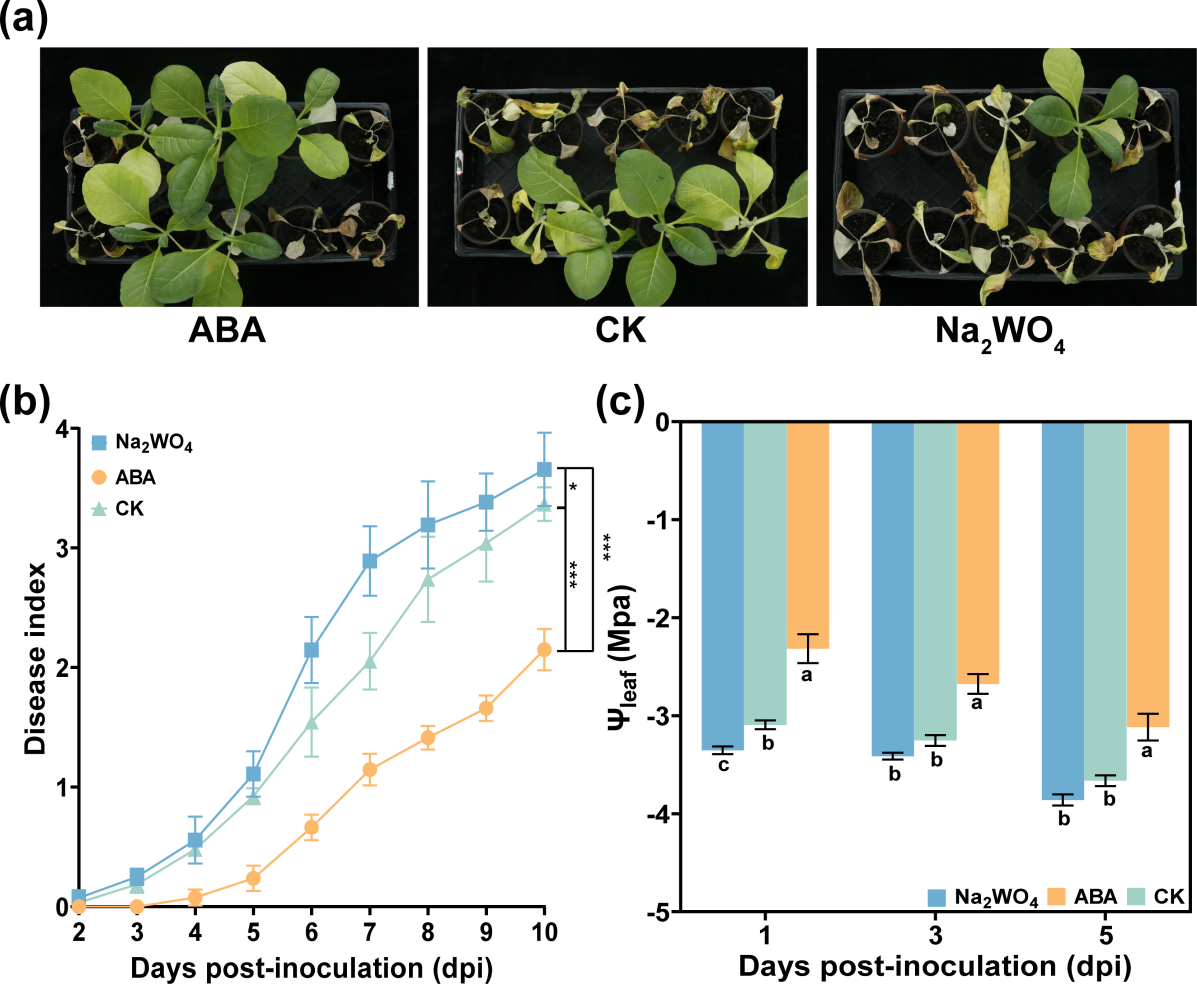
Effect of Na_2_WO_4_ on tobacco bacterial wilt and Ψ_leaf_ after *R. solanacearum* inoculation. **(a)** Occurrence of tobacco bacterial wilt at 9 dpi. **(b)** Disease index of tobacco bacterial wilt after Na_2_WO_4_ and CK (DI water) treatment. **(c)** Variations of Ψ_leaf_ after Na_2_WO_4_ treatment. The data are shown as mean ± SD. An asterisk (*) indicates a statistically significant difference between ABA and CK by *t*-tests. *p < 0.05, **p < 0.01, *** p<0.001. ns, not significant. The bars with lowercase letters indicate statistically significant differences by Tukey’s test (*p* < 0.05).

To further investigate whether ABA regulated Ψ_leaf_ and how it affected DI, DI and Ψ_leaf_ were measured after foliar spraying Na_2_WO_4_. As shown in **Figure 6a**, it was evident that the DI of Na_2_WO_4_ treatment was higher than that of CK at 3–13 dpi. In other words, when ABA synthesis in plant leaves was inhibited, the tobacco plant was more susceptible to infection by *R. solanacearum*, leading to a more serious incidence of the disease (**Figure 6b**). Interestingly, the Ψ_leaf_ of Na_2_WO_4_ treatment was also lower than that of CK and ABA treatments (**Figure 6c**). This result further confirmed that ABA may increase Ψ_leaf_ to delay the occurrence of the disease.

### 3.7 ABA promoted tobacco growth

**Figure 7:**
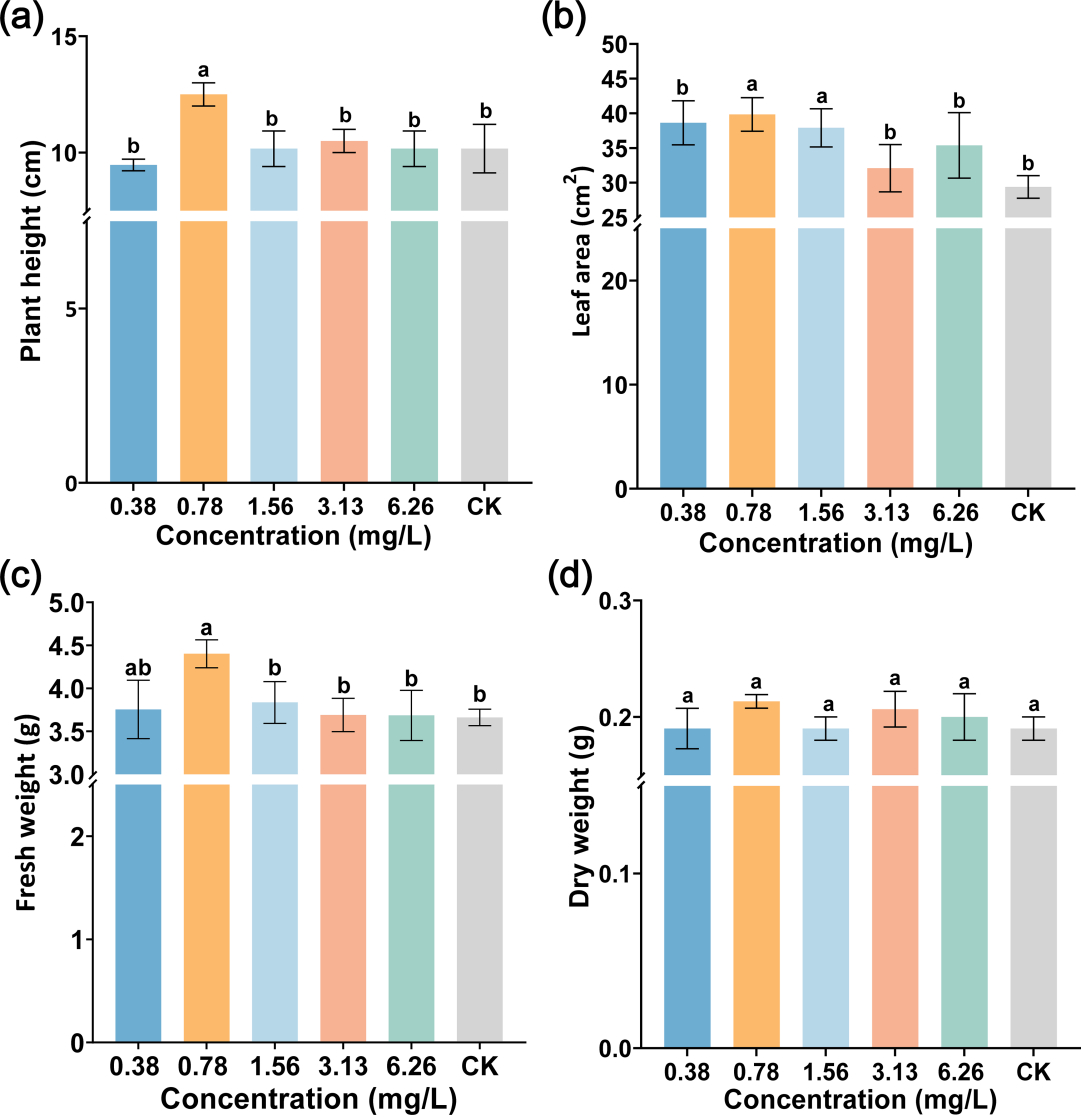
The effect of foliar spraying ABA on tobacco growth. **(a)** Plant height, **(b)** leaf area, **(c)** fresh weight, and **(d)** dry weight. The data are shown as mean ± SD. The bars with lowercase letters indicate statistically significant differences by Tukey’s test (*p* < 0.05).

To further analyze the role of ABA application at varied concentrations (0.38, 0.78, 1.56, 3.13, and 6.26 mg/L) on plant growth, at 15 days after spraying ABA, leaf area, plant height, and fresh and dry weight were investigated. As displayed in **Figure 7**, ABA did not inhibit tobacco plant growth. On the contrary, spraying 0.78 mg/L ABA could promote plant growth. The fresh weight, plant height, and leaf area with 0.78 mg/L ABA were 4.40 g, 12.50 cm, and 39.83 cm^2^, respectively, which were significantly higher than those of CK (3.66 g, 10.17 cm, and 29.39 cm^2^) as shown in **Figures 7a, b**. In addition, 0.78 mg/L ABA increased the fresh (or dry) weight by 20.21% (or 12.28%) compared with CK as shown in **Figures 7c, d**. Our study found that 0.78 mg/L ABA had a beneficial effect on promoting plant growth.

### 3.8 ABA reduced *R. solanacearum* infection in plants by reducing its mobility

**Figure 8:**
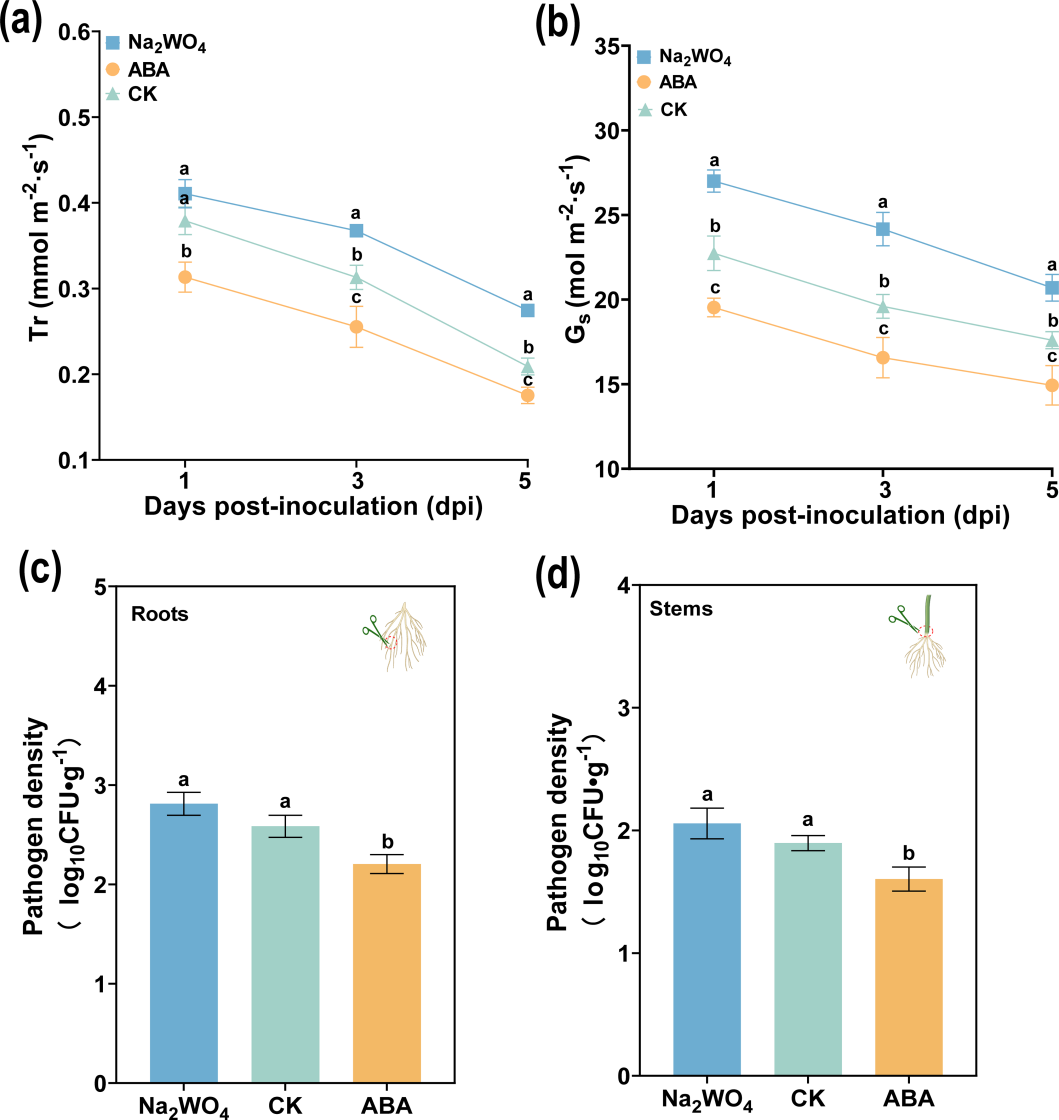
Effects of ABA on stomatal opening degree and transpiration rate of leaf, and bacterial population of *R. solanacearum* infection in tobacco. **(a)** Transpiration rate (Tr) and **(b)** stomatal conductance (Gs) of tobacco at 1, 2, and 3 dpi. **(c, d)** The pathogen density in the root **(c)** and stem base **(d)** after 3 dpi. The data are shown as mean ± SD. The bars with lowercase letters indicate statistically significant differences by Tukey’s test (*p* < 0.05).

Pn and Ci are important indicators of plant photosynthesis. To confirm that ABA regulates transpiration pull and plant photosynthesis, we tested the changes in Gs, Tr, Pn, and Ci after ABA and Na_2_WO_4_ application. As shown in **Figures 8a, b**, the Tr and Gs of all treatments decreased from 1 to 5 dpi, and the Tr and Gs in ABA-treated leaves were significantly lower than that with Na_2_WO_4_ and CK at 1, 3, and 5 dpi. In the long-term (or multiple) pot experiments, we found that the tobacco seedlings showed obvious wilting symptoms at 3 dpi. In this period, the Tr and Gs of ABA were 16.57 mmol m^-2^ s^-1^ and 0.26 mol m^-2^ s^-1^, which were significantly lower than those of CK (19.17 mmol m^-2^ s^-1^ and 0.31 mol m^-2^ s^-1^) and Na_2_WO_4_ (24.17 mmol m^-2^ s^-1^ and 0.37 mol m^-2^ s^-^). In addition, Pn and Ci (except for 5 dpi) also had similar results as shown in **Supplementary Figures S1a, b**, and Pn and Ci, related to photosynthesis after ABA application, were also significantly lower than those of CK and Na_2_WO_4_ at 1 and 3 dpi. At the same time, we further determined the infection of *R. solanacearum* in different tissues. As anticipated in **Figures 8c, d**, ABA treatment significantly reduced *R. solanacearum* infection compared to CK (DI water) and Na_2_WO_4_ at 3 dpi. The populations of *R. solanacearum* in root after ABA, CK, and Na_2_WO_4_ treatments were 2.30 × 10^5^ CFU/g, 2.78 × 10^5^ CFU/g, and 2.65 × 10^5^ CFU/g, respectively. Interestingly, compared to CK and Na_2_WO_4_ treatments, the populations of *R. solanacearum* in stem after ABA treatment were the lowest and reduced by 8.15% and 10.33%. The results also proved that ABA reduced the infection of *R. solanacearum* by decreasing the transpiration pull.

### 3.9 ABA improved defense enzyme activities

**Figure 9:**
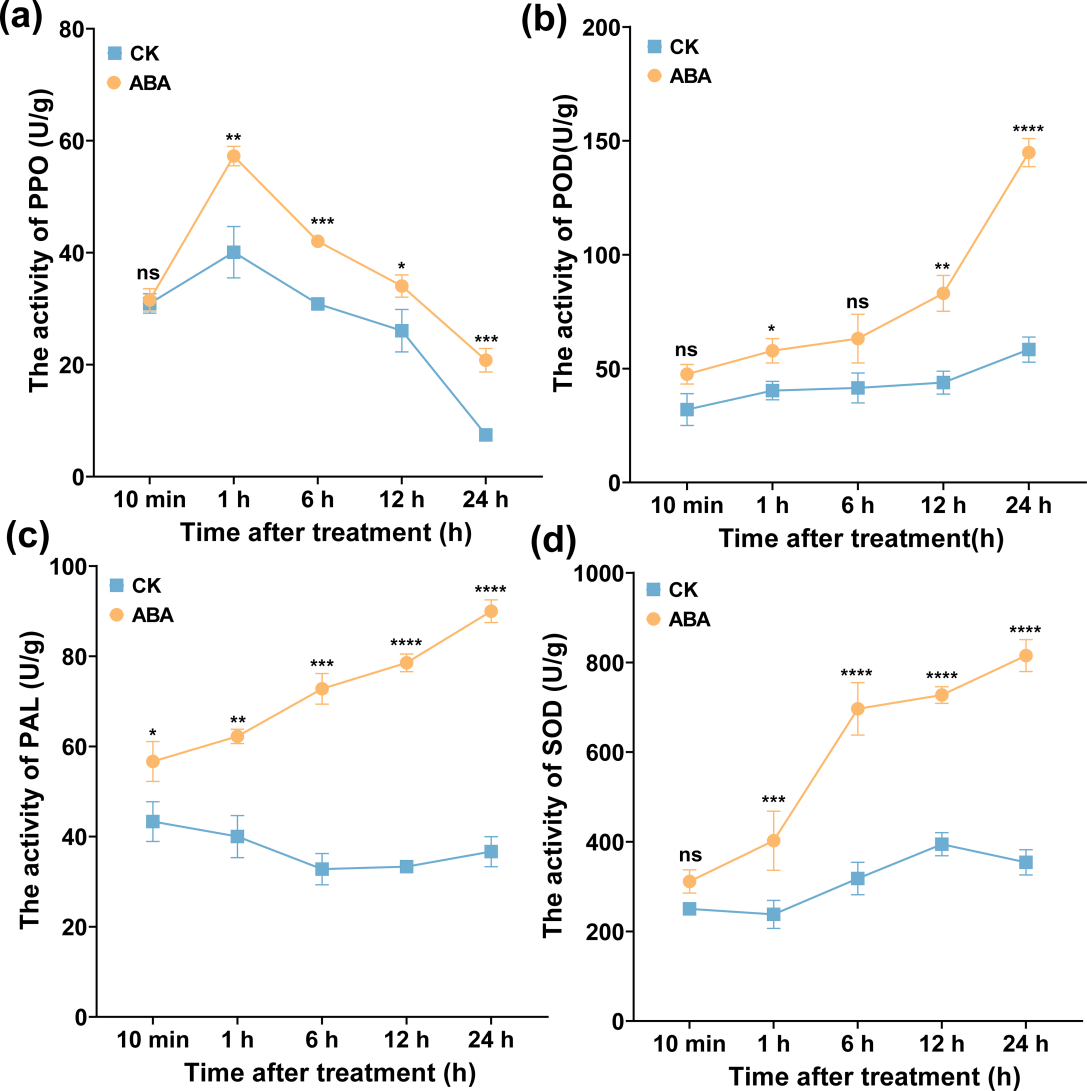
Effect of ABA on leaves’ defense enzymes. **(a)** PPO, **(b)** POD, **(c)** PAL, and **(d)** SOD, respectively. The data are shown as mean ± SD. An asterisk (*) indicates a statistically significant difference between the ABA and CK by *t*-tests. *p < 0.05, **p < 0.01, ***p < 0.001, ****p < 0.0001. ns, not significant.

To clarify whether ABA enhances plant resistance to *R. solanacearum* by activating the plant defense enzyme, PAL, POD, SOD, and PPO activities were measured. As displayed in **Figure 9**, 0.78 mg/L ABA increased the activities of PAL, POD, SOD, and PPO enzymes. The PPO activity showed a different trend with PAL, SOD, and POD activities (**Figure 9a**), which tended to increase first and then decrease within 24 h and peaked at 1 h. The PPO activity was at 57.25 U/g, which was significantly higher than CK (40.09 U/g). Notably, the activities of POD, PAL, and SOD showed a rising trend and peaked at 24 h (**Figures 9b–d**), which were 144.83, 89.99, and 815.43 U/g, respectively, significantly higher than CK (40.09, 36.66, and 354.00 U/g), and were increased by 2.61, 1.45, and 1.30 times, respectively. These results showed that foliar spraying ABA could activate the activities of plant defense enzymes.

### 3.10 ABA induced the expression of defense-associated genes

**Figure 10:**
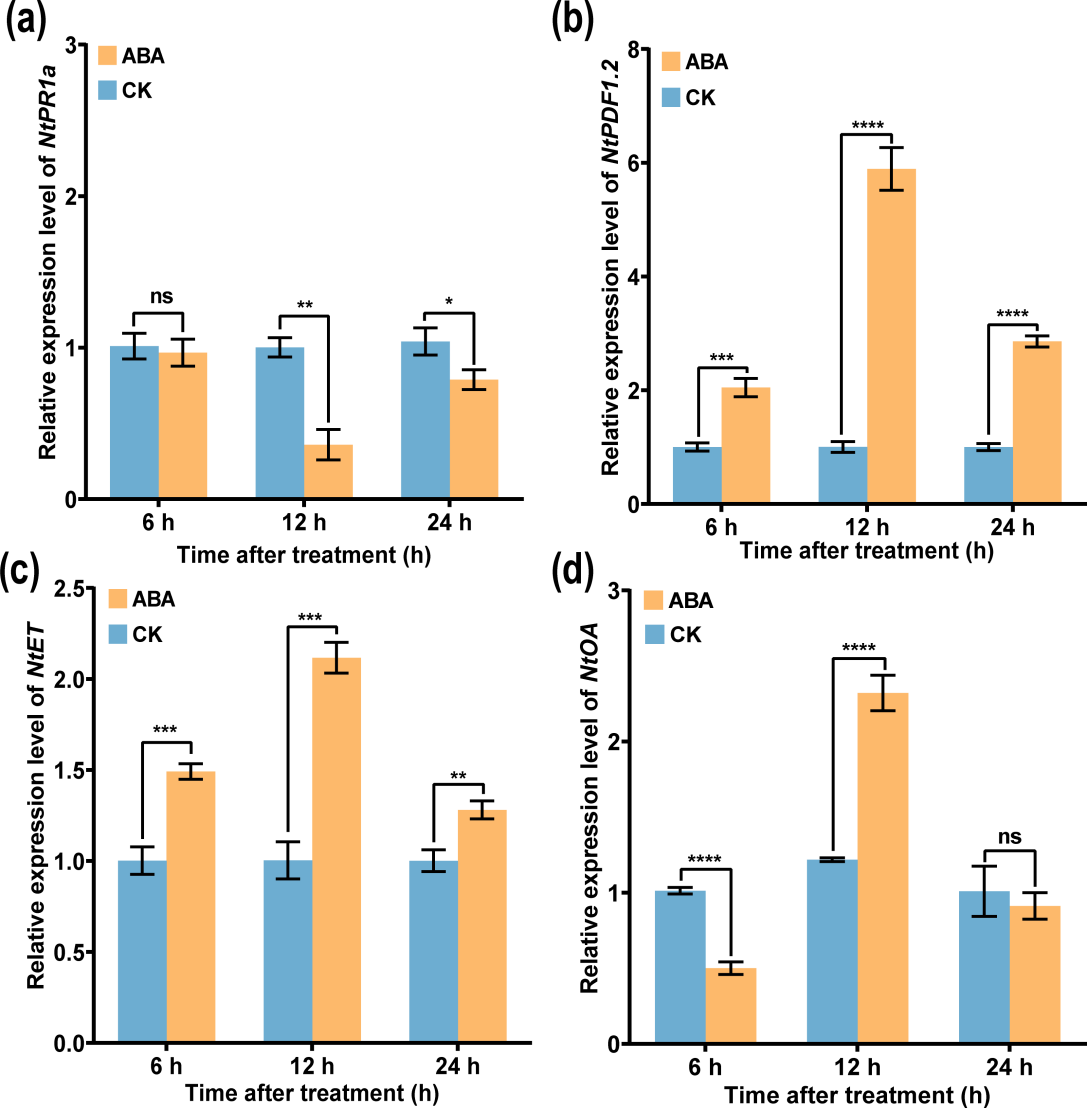
Expression of tobacco resistance genes induced by ABA. Relative expression of **(a)** *NtPR1*, **(b)** *NtPDF1.2*, **(c)** Nt*ET*, and **(d)** *NtOA*, respectively. An asterisk (*) indicates a statistically significant difference between the ABA and CK by *t*-tests. The data are shown as the mean ± SD. *p < 0.05, **p < 0.01, ***p < 0.001, ****p < 0.0001. ns, not significant.

To further analyze the underlying mechanism of ABA in inducing tobacco resistance, expression of defense-associated genes at different time points (6, 12, and 24 h) after ABA application was measured by using qRT-PCR. As shown in **Figure 10**, compared with CK, the expression levels of JA-related gene *NtPDF1.2*, ET synthesis gene *NtET*, and ROS-related gene *NtOA* increase first and then decrease from 6 to 24 h after spraying ABA, while the expression level of SA-related gene *NtPRa1* was significantly downregulated. In addition, the expression levels of *NtPDF1.2*, *NtET*, and *NtOA* genes were significantly upregulated compared with those of CK. Moreover, the expression level of *NtPR1a* gene after ABA application was significantly inhibited at 12 h (**Figure 10a**), and the expression level of CK was 1.72 times that of ABA, while the expression levels of *NtPDF1.2*, *NtET*, and *NtOA* genes were significantly upregulated, peaking at 12 h (**Figures 10b–d**), and were 3.59, 1.63, and 1.10 times, respectively, greater than those of CK. The results showed that ABA enhanced the stress resistance and defense ability of plants mainly by the JA/ET signaling pathway and the expression of ROS-related genes.

## 4 Discussion

It is well known that the leaf stomata could alleviate the symptom by reducing plant water loss. ABA widely regulated plants for the uptake and utilization of water in conditions of water deprivation (Du et al., 2018; Kuromori et al., 2018). In particular, ABA was also utilized in crop production and disease management. In addition, it was widely known that ABA played a part in regulating plant stomatal closure (Meigas et al., 2024; Yoshida and Fernie, 2018). Its action pathway (Lim et al., 2015) and signaling mechanism (Hsu et al., 2021) had been previously studied. In this study, we discovered that PWC and plant water potential gradually decreased after *R. solanacearum* infection, while the stomatal size, Gs, and Tr decreased first and then increased. Notably, ABA content increased first and then decreased, which indicated that ABA was involved in the regulation of Ψ_leaf_, Ψ_stem_, and Ψ_root_ after the process of *R. solanacearum* infection. In addition, foliar spraying ABA could reduce plant water loss by reducing Gs and Tr, a finding which was similar to previous research (Li et al., 2022; Lim et al., 2015). Interestingly, the Ψ_leaf_, Ψ_stem_, and Ψ_root_ decreased from 24 to 96 h, which may be between grades 1 and 2, and the grade may exhibit greater water mobility and have a lower water potential. Similarly, at higher levels of ABA, the stomatal size, stomatal aperture, and leaf area decreased, which reduced the leaf Tr and improved the water utilization efficiency (Zhang et al., 2018). Water potential was a physiological index to measure water balance in plants (Tao et al., 2016; Wang et al., 2022). A previous study found that change of water potential promoted the accumulation of ABA primarily in the leaves, and ABA primarily enhances drought resistance in tomato by acting on leaf stomata rather than on the xylem (Haverroth et al., 2023). This study found that the Ψ_leaf_ and ABA content, respectively, were significantly higher in K326 than in Yunyan 87. The Ψ_leaf_ of tobacco plants (K326 and Yunyan 87) was increased and regulated by ABA after *R. solanacearum* infection. Furthermore, the application of Na_2_WO_4_, an inhibitor of ABA synthesis, promoted the occurrence of tobacco bacterial wilt, and spraying 0.78 mg/L ABA could delay the occurrence of the disease. These results indicated that ABA enhanced plant resistance to *R. solanacearum*, consistent with previous findings reported by Yop et al. (2023). Conversely, Zhou et al. reported a negative regulation of ABA to *R. solanacearum* in tobacco (Zhou et al., 2008), which may be related to the concentration of ABA. Zhou et al. used 50 μM ABA (about 0.013 mg/L), while we used 0.78 mg/L. In a previous indoor experiment, we observed significant differences in the effect of ABA on tobacco’s resistance to *R. solanacearum*. Specifically, disease incidence increased with 12.52 mg/L of ABA but decreased with 0.78 mg/L of ABA (**Supplementary Figure S2**). Furthermore, the ability of ABA to activate plant resistance was related to the concentration of ABA application.

The vital role played by ABA in plant growth and development is increasingly emphasized. Studies have shown that ABA impacted plant growth to a certain extent, and plants lacking ABA exhibit delayed growth, a phenomenon associated with ABA concentration (Brookbank et al., 2021). In addition, the exogenous application of ABA promoted rice root hair elongation by regulating auxin homeostasis (Wang et al., 2021). We also investigated the effect of ABA on tobacco plant growth. The results revealed that 0.78 mg/L ABA had a positive role in increasing plant height, leaf area, and fresh (or dry) weight. In the aspect of plant disease management, in the interaction between host plant and pathogenic bacteria, plant hormones (ABA, SA, and JA) mediated the plant immunity. A past study had demonstrated that ABA could promote JA synthesis and inhibit SA synthesis after pathogenic bacteria invasion (Long et al., 2019). In addition, a study on the involvement of ABA in JA signal transduction in *Arabidopsis* found that there was a close relationship between ABA and JA, and ABA receptors participated in JA signal transduction through *JAZ*, *MYC2*, and *COI* transcript regulation (Yu et al., 2021). Similarly, in our study, we found that spraying ABA at 0.78 mg/L could upregulate the expression levels of JA-related gene *NtPDF1.2* and repress the expression levels of SA-related gene *NtPR1a*. Interestingly, the expression levels of ET synthesis gene *NtET* and ROS-related gene *NtOA* upregulate after ABA treatment, which showed that ABA improved the tobacco plant’s resistance by activating the synergistic regulation of JA, ET, and ROS signaling pathways. However, another study found that JA and ABA were antagonistic after infection with pathogenic bacteria (Du et al., 2024; Han et al., 2023), which was different from our results. The concentration of ABA may have changed after exogenous application, leading to subsequent changes in physiological and biochemical mechanisms.

**Figure 11:**
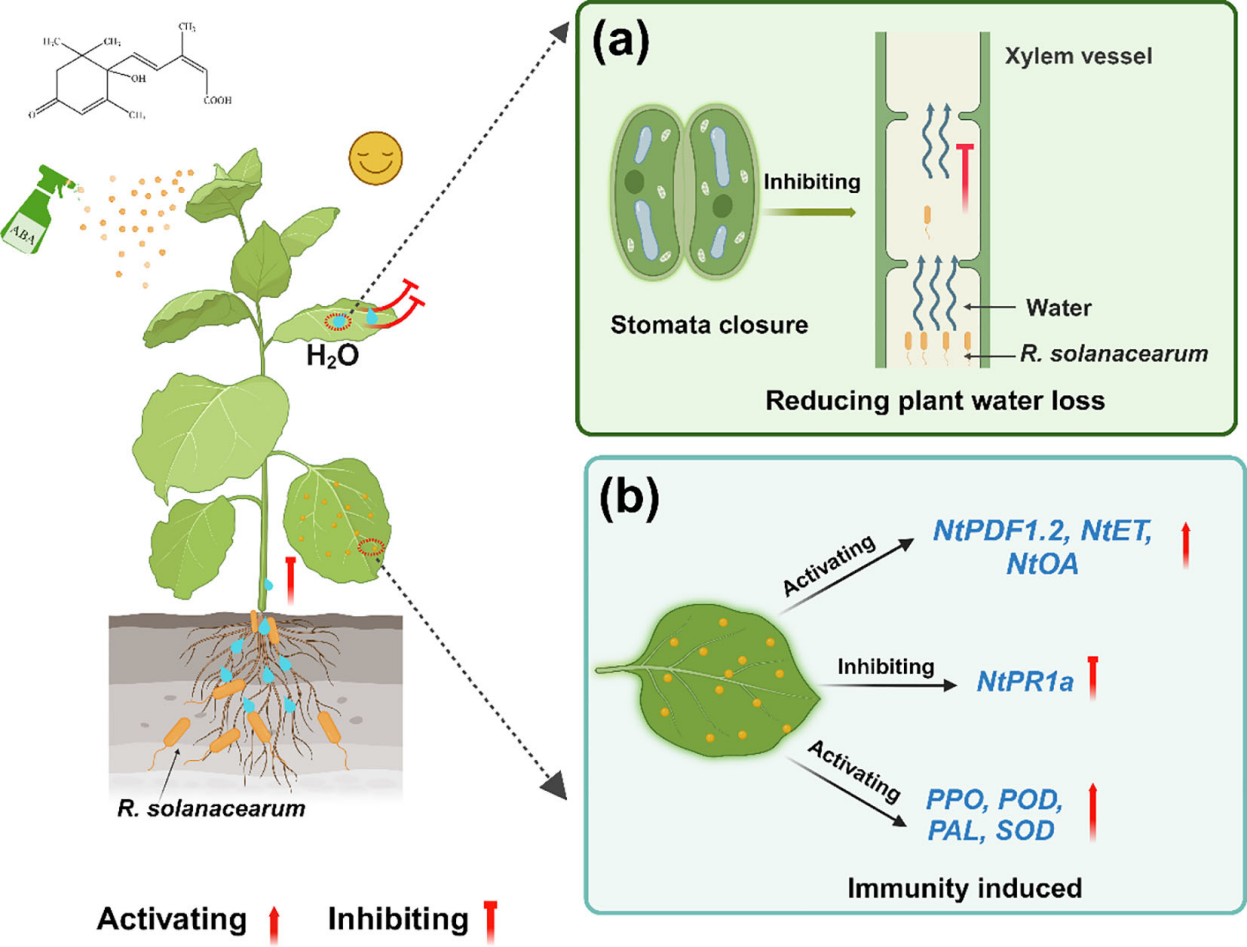
A model of foliar spraying ABA alleviated tobacco bacterial wilt. **(a)** Reducing plant water loss. **(b)** Immunity induced.

Few studies report that 0.78 mg/L ABA could delay the occurrence of tobacco bacterial wilt. We concluded that foliar spraying ABA alleviated the wilting caused by *R. solanacearum* by reducing plant water loss and enhancing the plant system’s resistance. To illustrate the role of ABA after R. solanacearum infection, a model of foliar spraying ABA alleviated tobacco bacterial wilt was proposed (**Figure 11**). ABA maintained the balance of water in the early stage (grades 0 to 1 or grades 0–2) of plants by reducing Gs and Tr, which slowed down *R. solanacearum* movement from the roots to the stems. Furthermore, ABA improved disease resistance by promoting plant growth, enhanced plant defense enzyme activities, and activated the expression of JA/ET pathway-related genes and ROS-related genes. These findings suggest the possible functions of low-concentration ABA. A follow-up research is required to clarify the specific mechanisms and causal links involved in these processes.

## 5 Conclusion

This study analyzed the preliminary mechanism of low-concentration ABA involved in *R. solanacearum* infection in tobacco plants. The results showed that PWC and Ψ_plant_ will decrease after *R. solanacearum* infection, and LWC and Ψ_leaf_ were decreased significantly. In response to biotic stress, plant leaves will accumulate a large amount of ABA to reduce the plant’s water loss. Interestingly, ABA accumulation in the leaves could enhance plant resistance to *R. solanacearum*, while inhibition of ABA biosynthesis promoted tobacco bacterial wilt. The application of 0.78 mg/L ABA reduced the colonization of *R. solanacearum*, and stimulated the activity of defense enzymes and resistance-associated genes. Moreover, the application of 0.78 mg/L ABA efficiently delayed the disease. This study clarifies initially the change of ABA-regulated plant water and induced plant resistance to tobacco bacterial wilt.

## Data Availability

The original contributions presented in the study are included in the article/**Supplementary Material**. Further inquiries can be directed to the corresponding authors.
