# Supplementary material for: Plants accumulate abscisic acid after Ralstonia solanacearum infection for enhanced dehydration tolerance and plant resistance
Source: Front Plant Sci. 2025 Jun 5;16:1566215. doi: 10.3389/fpls.2025.1566215 (PMC12176866; doi:10.3389/fpls.2025.1566215)
Supplement: Supplementary file 1 [file DataSheet1.docx]

**Supporting Information**

**Supplementary experimental details**

**Table S1. Primers related to disease resistance genes**

| **Gene name** | **Forward primer** | **Reverse primer** |
| --- | --- | --- |
| *NtPDF1.2(Wang et al. 2024)* | GGAAATGGCAAACTCCATGCG | ATCCTTCGGTCAGACAAACG |
| *NtPR1a(Wang et al. 2024)* | TCCACACTCGCCATCCTGAA | GTGCCGCAAATTCAACTCTG |
| *NtET(Tang et al. 2017)* | AGCTTCCCTTTATGCCCAGA | ACTGTTGTTACATCGCACCCT |
| *NtOA* | GGCCCCATTGGAACGATGTA | GACAGACCAACGCACGAAAC |
| *NtGAPDH* | GGTGTCAAGCAAGCCTCTCA | GGCTCCAGCCTGAATGTGT |

Note: *NtPDF1.2* represents key genes of jasmonic acid pathways; *NtPR1a* represents key genes of *salicylic acid* pathway; *OA* represents ROS related genes; *ET* represents the salicylic acid pathway regulatory gene; *NtGAPDH* represents reference gene.


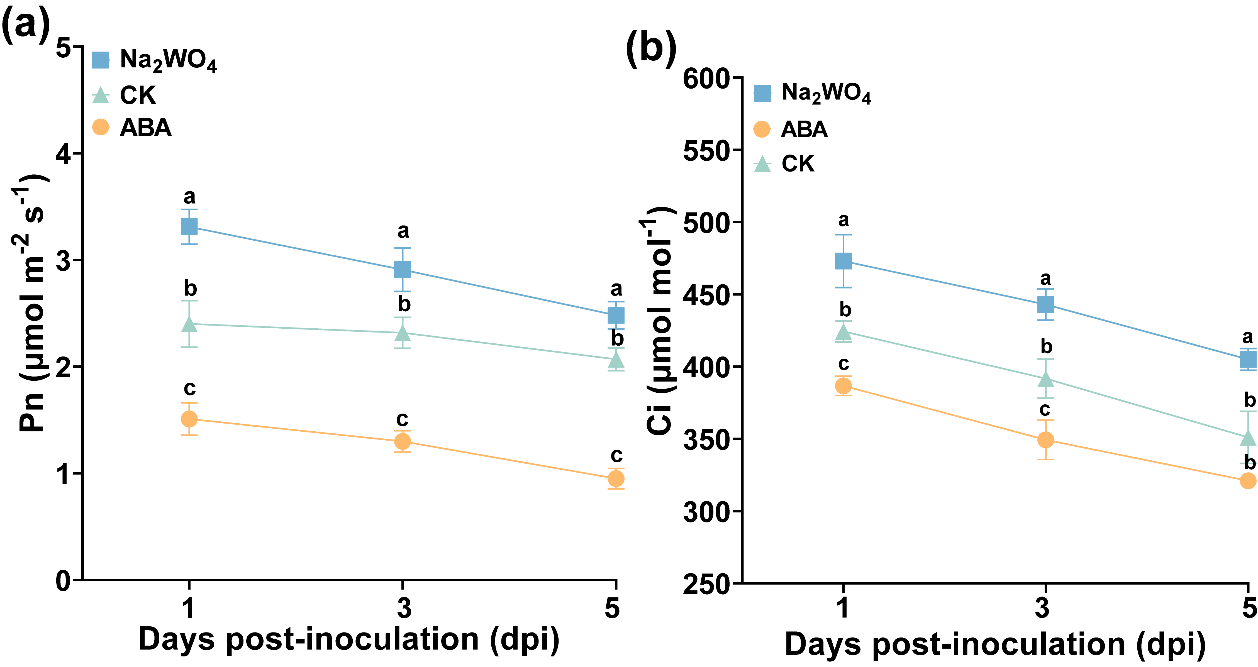


**Fig.S1.** **The effect of foliar spraying ABA on plant photosynthesis.** (a) Net photosynthetic rate (Pn), (b) Intracellular CO_2_ concentration (Ci). The data are shown as the mean ± SD of six replicates each comprising six plants. The bars with lowercase letters (b) indicate statistically significant differences by Tukey (*p < 0.05*).

**
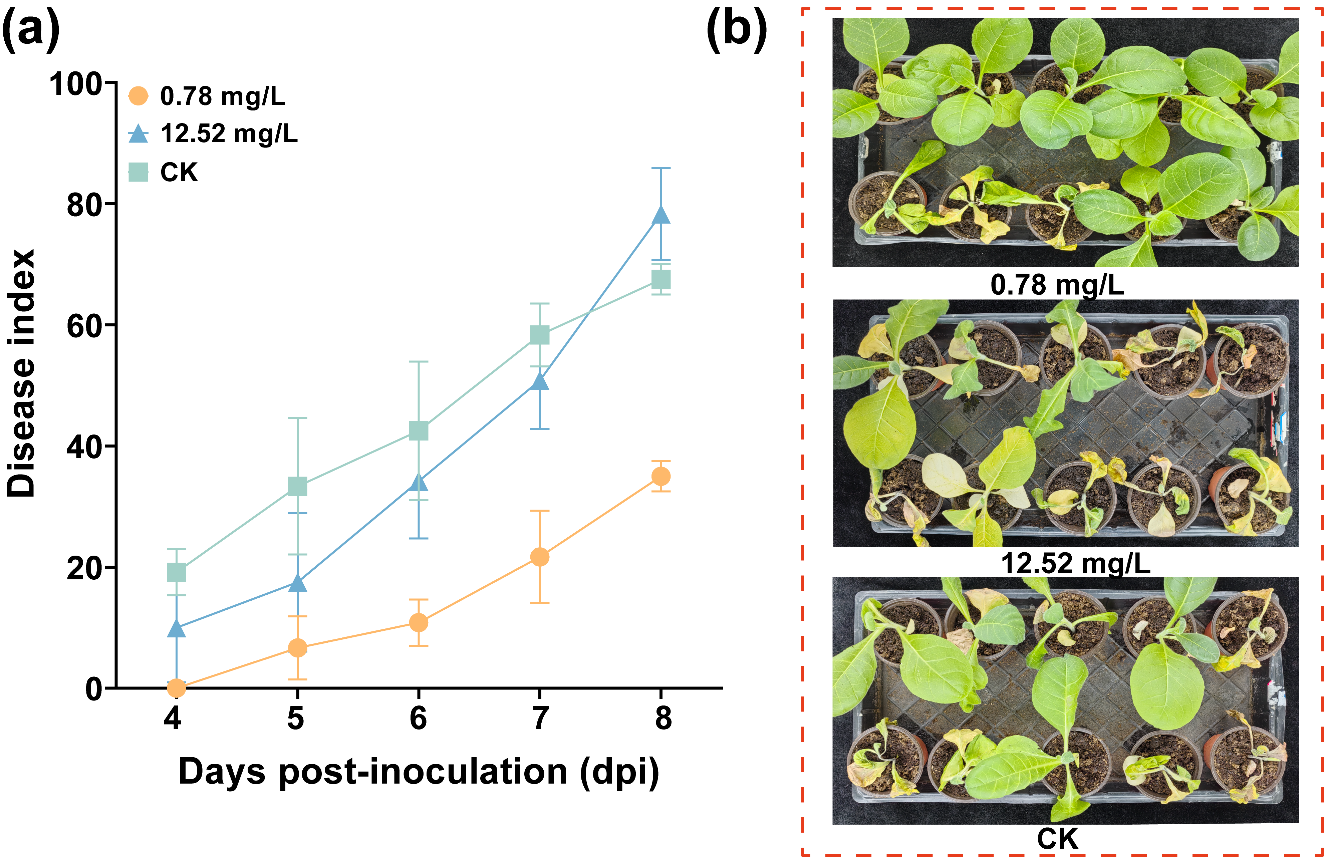
**

**Fig.S2.** **Control efficiency of different concentrations ABA on tobacco bacterial wilt.** (a) The disease index after treatment with 0.78 mg/L ABA, 12.52 mg/L ABA, and CK (DI water), (a) The occurrence of tobacco bacterial wilt at 8 dpi.

**References**

Tang Y, Liu Q, Liu Y, Zhang L, Ding W. (2017). Overexpression of NtPR-Q up-regulates multiple defense-Related genes in *Nicotiana tabacum* and enhances plant resistance to *Ralstonia solanacearum*. *Front. Plant Sci.* 8. https://doi.org/10.3389/fpls.2017.01963.

Wang Y, Liang Y, Ma X, Dong Y, Klakong M, Wang A, et al. (2024). An environmental-friendly resistance inducer in crop protection: V2C MXene nanosheets induce plant resistance to Ralstonia solanacearum via the ET/JA signaling pathway. *Ind. Crop. Prod.* 220, 119269. <https://doi.org/10.1016/j.indcrop.2024.119269>.
